# C9C5 positive mature oligodendrocytes are a source of Sonic Hedgehog in the mouse brain

**DOI:** 10.1371/journal.pone.0229362

**Published:** 2020-02-20

**Authors:** Linda Tirou, Mariagiovanna Russo, Helene Faure, Giuliana Pellegrino, Ariane Sharif, Martial Ruat

**Affiliations:** 1 UMR-9197, Neuroscience Paris-Saclay Institute, CNRS, Paris Saclay University, Gif-sur-Yvette, France; 2 UMR-S Inserm 1172/Lille University, France; Massachusetts General Hospital/Harvard Medical School, UNITED STATES

## Abstract

In the mature rodent brain, Sonic Hedgehog (Shh) signaling regulates stem and progenitor cell maintenance, neuronal and glial circuitry and brain repair. However, the sources and distribution of Shh mediating these effects are still poorly characterized. Here, we report in the adult mouse brain, a broad expression pattern of Shh recognized by the specific monoclonal C9C5 antibody in a subset (11–12%) of CC1^+^ mature oligodendrocytes that do not express carbonic anhydrase II. These cells express also Olig2 and Sox10, two oligodendrocyte lineage-specific markers, but not PDGFRα, a marker of oligodendrocyte progenitors. In agreement with oligodendroglial cells being a source of Shh in the adult mouse brain, we identify Shh transcripts by single molecule fluorescent *in situ* hybridization in a subset of cells expressing Olig2 and Sox10 mRNAs. These findings also reveal that Shh expression is more extensive than originally reported. The Shh-C9C5-associated signal labels the oligodendroglial cell body and decorates by intense puncta the processes. C9C5^+^ cells are distributed in a grid-like manner. They constitute small units that could deliver locally Shh to its receptor Patched expressed in GFAP^+^ and S100β^+^ astrocytes, and in HuC/D^+^ neurons as shown in Ptc^LacZ/+^ reporter mice. Postnatally, C9C5 immunoreactivity overlaps the myelination peak that occurs between P10 and P20 and is down regulated during ageing. Thus, our data suggest that C9C5^+^CC1^+^ oligodendroglial cells are a source of Shh in the mouse postnatal brain.

## Introduction

Sonic Hedgehog (Shh) is a secreted molecule implicated in neural patterning during embryogenesis. It acts at short- and long-range through its release as soluble multimers and its transport in extracellular particles or along filopodia [1]. In the mature rodent brain, the protein is implicated in stem cell maintenance and brain repair, in the plasticity of neuronal circuits and in mediating communication between neurons and astrocytes. Shh is autoproteolytically cleaved to an amino-terminal active fragment (ShhN), which is widely distributed in the rodent brain. Its axonal transport suggests that the protein, which is synthetized by a restricted population of GABAergic, cholinergic, and layer V corticofugal neurons, can signal at the nerve terminals [2, 3]. Shh binds to the transmembrane receptor Patched (Ptc), which relieves the repression exerted on the G-protein coupled receptor Smoothened. This initiates signal transduction implicating the activation of the transcription factors of the Gli family (Gli1-3) and the transcription of targets genes [1]. However, several non-canonical pathways not linked to gene transcription have been described [4].

Activation of Shh signaling in astrocytes has been proposed to promote neuroprotection [5], to reduce neuronal activity [6, 7] and to keep their physiology [8] whereas its attenuation leads to reactive astrocytosis [9]. Shh from neuronal sources [10, 11, 12, 13, 14, 15] regulates adult mouse neurogenic niches providing olfactory bulb interneurons and granule neurons in the dentate gyrus of the hippocampus. Shh present in the cerebrospinal fluid [16] may regulate the neurogenic niche in the subventricular zone of the lateral ventricle and the balance between quiescence and activation of neural stem cells [17]. Shh signaling also participates in the embryonic and postnatal production of oligodendroglial cells and is of interest for remyelination [18].

Here, we investigated the distribution pattern of Shh in the mouse brain using the monoclonal C9C5 antibody directed against human SHHN. We further characterized a broad distribution of C9C5 immunoreactive signal in brain tissues, identified a subset of mature oligodendrocytes as a source of Shh and showed that these cells constitute Shh-expressing domains in the brain.

## Materials and methods

### Animals

Post-natal and adult C57BL/6J male mice were from Janvier Labs (Genest-Saint-Isle, France). Glast-Cre^ERT2^R26R-YFP and Ptc^+/LacZ^ mouse lines have been described [19]. Adult mice (10 weeks or older as indicated) were used. Animal experiments were performed in accordance with the Council Directive 2010/63EU of the European Parliament and approved (project n°4558) by the French ethic committee (C2EA– 59 Comite Paris Centre et Sud).

### Cell culture and transfection

HEK293 (ATCC) cells were cultured, transfected using X-tremeGENE9 (Sigma) with pRK5-mShh for the expression of mouse Sonic Hedgehog (mShh) (provided by Dr P. Beachy) or empty vector and harvested 48h later as described [10].

### Western blotting

Adult brain tissues from four mice were dissected, homogenized in RIPA Buffer (Thermo) and solubilized (1h30). Twenty μg of the cleared tissues supernatants and 1 μg of mock or mShh transfected HEK293 homogenates were subjected to SDS-PAGE and Western blotting [10]. Protein concentrations were determined using the BCA protein assay (Thermo, #23225). Anti-SHHN C9C5 rabbit monoclonal antibody (1/1000, #2207, Cell Signaling, epitope surrounding Glu53 of human SHH), and anti-Actin AC-40 mouse monoclonal antibody (1/2000, #A4700, Sigma) were probed overnight and chemiluminescence was acquired with Chemidoc apparatus (BIORAD). The specificity of the C9C5 antibody was assessed by Western blot in three independent blocking experiments: C9C5 antibody was pre-incubated overnight at 4°C with 1 or 30 μg/ml of mouse Shh (residues 25–198) fused to glutathione-S-transferase (GST-mShhN) or of GST alone [10] for brain extracts and cell homogenates, respectively. Specificity of the C9C5 antibody for ShhN in brain extracts is also provided in S1 Fig.

### Immunofluorescence

Mice were deeply anesthetized with intraperitoneal injection of Xylazine (Rompun®, 10 mg/kg) and Ketamine (Imalgène®, 100 mg/kg) and were perfused with 4% paraformaldehyde (PFA) in 0.2 M sodium phosphate, pH 7.4. After dissection, brains were post-fixed for 2 h in 4% PFA and cryoprotected in 10% sucrose in PBS until tissue sank (6–12 hrs) and then 30% sucrose in PBS until tissue sank. The tissues were embedded in Optimal Cutting Temperature (O.C.T.) compound Tissue-Tek (Sakura Finetek), frozen in liquid nitrogen cooled isopentane and kept at -80°C until use. Tissues were allowed to equilibrate to –20°C in a cryostat (Leica, CM3050) for ~30 min and 14 μm coronal sections were prepared and mounted onto SuperFrost® ultra Plus slides (Thermo Scientific). For immunostaining, sections were incubated 1h in PBS, 0.25% Triton, 1% BSA and 1h with donkey anti-mouse Fab fragment (1/50, 715-007-003, Jackson IR). The primary antibodies were incubated overnight at 4°C: rabbit anti-SHHN (1/300, C9C5, #2207, Cell Signaling), mouse anti-GFAP (1/400, MAB360, Millipore), goat anti-Olig2 (1/400, AF2418, R&D Systems), mouse anti-S100β (1/500, S2532, Sigma), mouse anti-adenomatous polyposis coli (APC) (1/600, clone CC1, OP80, Millipore), chicken anti-βgalactosidase (1/200, ab9361, Abcam), goat anti-Sox10 (1/100, AF2864-SP, R&D Systems), rat anti-PDGFRα (1/300, 558774, BD Pharmingen), sheep anti-Carbonic Anhydrase II (CAII) (1/200, AHP206, BIORAD), rabbit anti-Iba1 (1/500, 019–19741, Fujifilm Wako), mouse anti-HuC/D (1/400, A-21271, Molecular Probes). Sections were incubated with the appropriate secondary antibody (1/200 to 1/400, Millipore, Jackson IR) for 2h at room temperature. Images were acquired with a 40X objective (N.A. 0.75) using fluorescence (Leica DM2000) or confocal (Leica TCS-SP8) microscope. For the later, Z-stacks (9.6 μm), containing twelve Z-sections images were analyzed using LASAF (Bitplane AG) and reconstructed in ImageJ 1.39t (Freeware, NIH) and Photoshop-CS3 (Adobe). C9C5 immunostaining was performed from the brain of twenty adult mice samples used in this study with similar results. The specificity of C9C5 antibody was assessed on adult mouse brain sections in three independent blocking experiments: C9C5 antibody was pre-incubated overnight at 4°C with 400 μg/ml of recombinant mouse ShhN fragment (residues 25–198) fused to glutathione-S-transferase (GST-mShhN) or of GST alone as control [10]. Single molecule fluorescent *in situ* hybridization was performed on frozen brain sections of three adult mice using the RNAscope® Multiplex Fluorescent Kit-v2 according to the manufacturer’s protocols (Advanced Cell Diagnostics, Newark). Specific probes were used to detect Shh (#314361), Olig2 (#447091-C2), Sox10 (#435931-C3) and GAD (#400951-C3) mouse mRNAs.

### Cell counting and statistical analysis

Quantification of C9C5^+^ cells in the hypothalamic parenchyma was performed on sections obtained at the level of the median eminence from three animals at P4, P10 and 9 months stainings, or four animals at P20, 2 and 12 months, and was expressed as a number of cells per mm^2^. The number of C9C5^+^ counted cells per animal ranged between 0 and 4 at P4, 45 and 57 at P10, 41 and 70 at P20, 39 and 61 at 2 months, 4 and 8 at 9 months, and 0 and 3 at 12 months. Quantification of C9C5^+^CC1^+^Olig2^+^, C9C5^+^CC1^+^Sox10^+^, C9C5^+^CAII^+^ and C9C5^+^PDGFRα^+^ co-staining in the cerebral cortex and hypothalamic parenchyma was performed on sections obtained at the level of the median eminence from three adult mice except for Sox10 staining (n = 4). The number of counted cells per region and per animal ranged between 200 and 350 for CC1^+^ cells, 300 and 700 for Olig2^+^ cells, 300 and 600 for Sox10^+^ cells, 100 and 250 for CAII^+^ cells, 50 and 200 for PDGFRα^+^ cells. Quantification of βgalactosidase^+^S100β^+^ co-staining was performed in the hypothalamic parenchyma at the level of the median eminence from four adult mice. The number of S100β^+^ counted cells per animal ranged between 250 to 325 cells. Quantification of *Shh*^*+*^*Olig2*^*+*^ and *Shh*^*+*^*Sox10*^*+*^ co-stainings in the cerebral cortex and in the hypothalamic parenchyma was done on sections obtained at the level of the median eminence from three adult mice. The number of counted cells per region and per animal ranged between 120 to 300 *Olig2*^*+*^ cells and from 100 to 550 *Sox10*^*+*^ cells. Countings of co-localised stainings were done using ROI and multi-point ImageJ tools. Quantitative data are expressed as the mean ± standard error of the mean (SEM). Comparisons between two experimental groups were made using unpaired Student’s t-tests. A value of p < 0.05 was considered statistically significant. Graphs were drawn using GraphPad Prism 5.2 (GraphPad Software, Inc).

## Results

### The Sonic Hedgehog antibody ‘C9C5’ labels CC1 positive oligodendrocytes

**Fig 1:**
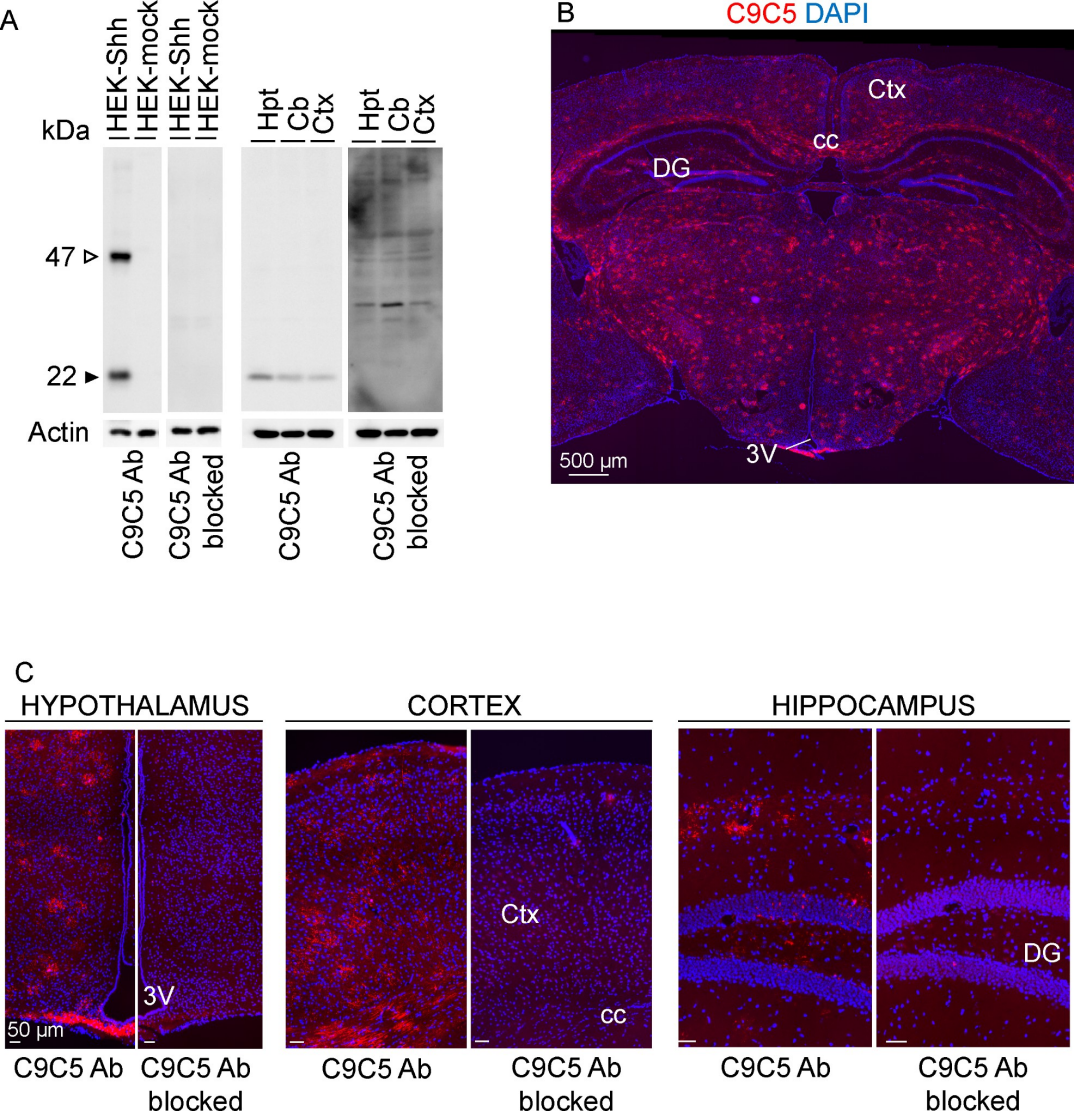
Characterization of the C9C5 anti-SHHN antibody expression pattern. (A) Western blot of protein homogenates from HEK293 cells transfected with a mouse Shh (HEK-Shh) or control vector (HEK-mock) and from hypothalamic (Hpt), cerebellar (Cb) or cerebrocortical (Ctx) tissues of an adult mouse with the C9C5 antibody, and was reproduced with tissues from three other animals. A 22 kDa band, the expected size for the aminoterminal fragment of Shh, was detected in HEK-Shh cells and in tissues but was absent in HEK-mock cells and in blocking experiments (n = 3). A 47 kDa signal corresponding to the uncleaved Shh protein was detected in HEK-Shh cells but not in tissues. Actin served as a loading control. (B-C) Low-magnification photomontages of coronal brain sections of adult mice showing strong C9C5 immunoreactivity in almost all brain regions (B), which was absent in blocking experiments (n = 3) (C). Staining was replicated in the twenty adult mice used in this study. Nuclei stained with DAPI. 3V, third ventricle; Hpt, Hypothalamus; Ctx, cerebral cortex; Cb, cerebellum; cc, corpus callosum; DG, dentate gyrus of the hippocampus.

We sought to identify in the adult mouse brain, the protein expression and cell distribution of Sonic Hedgehog (Shh) using the monoclonal ‘C9C5’ antibody directed against the amino-terminal fragment of human SHH. From Western blot experiment analysis (Fig 1A, S1 Fig), we observed that the C9C5 antibody detected a 22 kDa signal corresponding to the expected size for the cleaved amino-terminal fragment of Shh (ShhN) in both HEK293 cells expressing mouse Shh and brain tissues lysates from adult mice. This signal was eliminated in blocking experiments (Fig 1A) consistent with this signal being related to ShhN.

**Fig 2:**
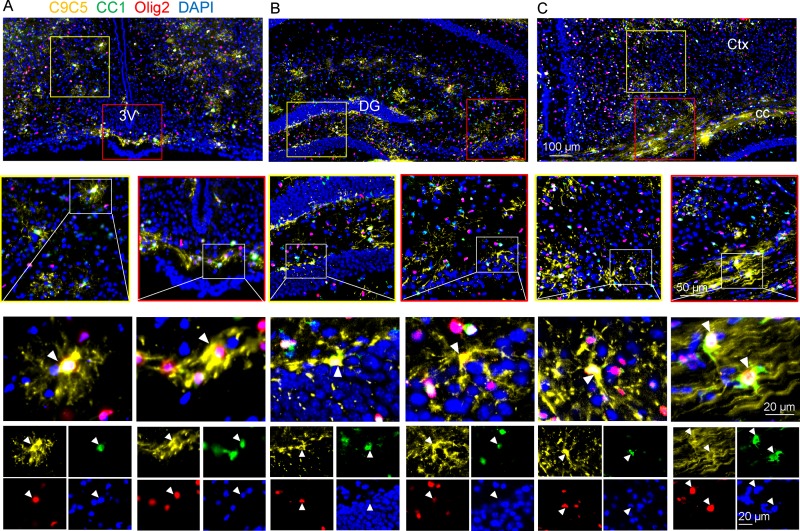
C9C5^+^ cells express the oligodendroglial markers CC1 and Olig2. (A-C) Immunostaining of coronal brain sections from an adult mouse of the hypothalamic median eminence (A), the hippocampus (B), the cerebral cortex and corpus callosum (C) with the C9C5 antibody (yellow) and the oligodendroglial markers CC1 (green) and Olig2 (red). Yellow and red squares highlight magnifications of the ventromedial hypothalamic nucleus and the median eminence (A), the dentate gyrus and CA3 pyramidal layer (B) of the hippocampus, the cerebral cortex and the corpus callosum (C). White boxes show C9C5/CC1/Olig2 triple positive cells with stellate morphology presented in merge and single channels with the nuclear marker DAPI. Staining was replicated at least on three mice. 3V, third ventricle; Ctx, cerebral cortex; cc, corpus callosum; DG, dentate gyrus of the hippocampus.

**Fig 3:**
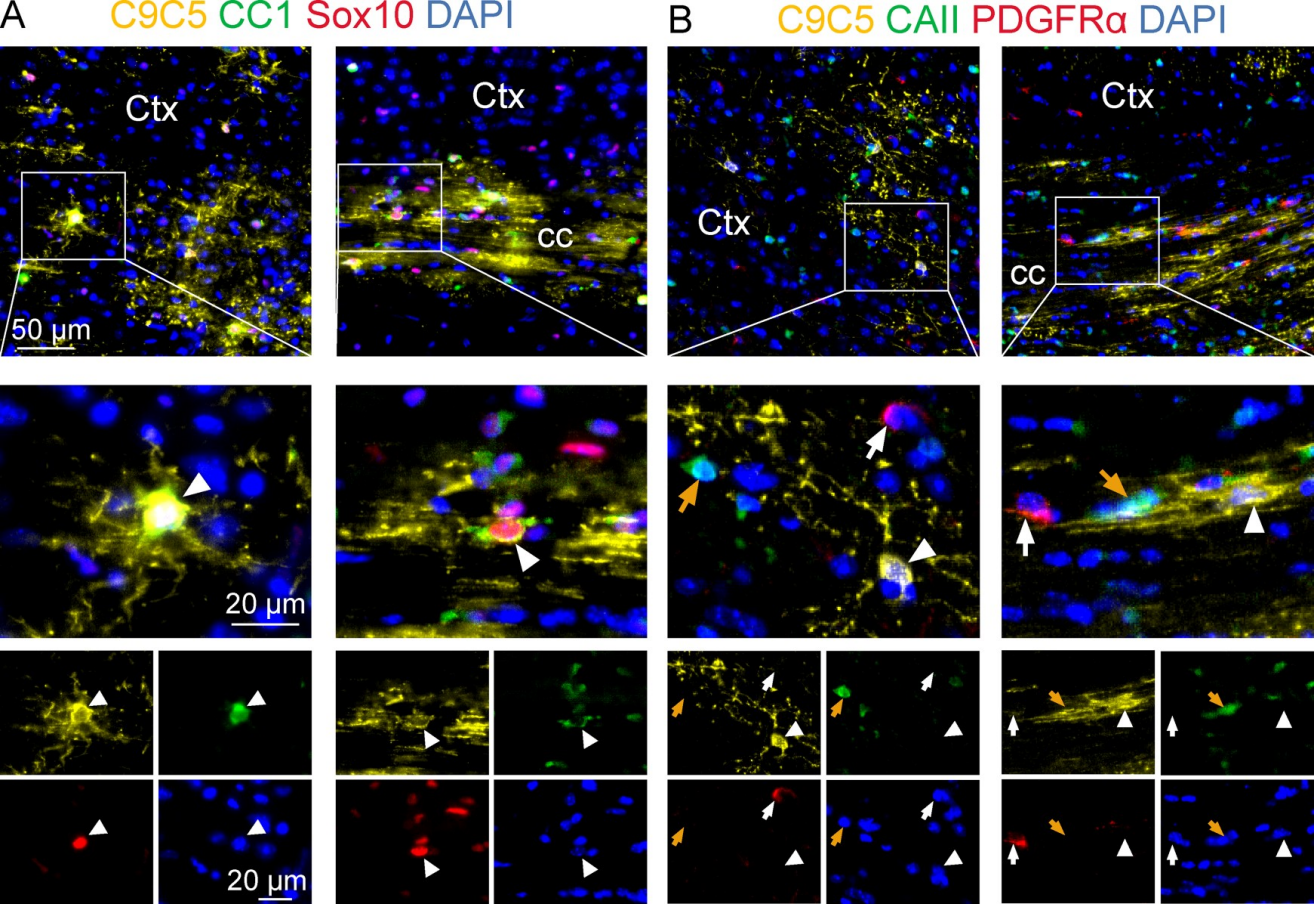
Characterization of C9C5^+^ cells at different stages of the oligodendroglial lineage. (A-B) Immunostaining of coronal brain sections from an adult mouse of the cerebral cortex and corpus callosum with the C9C5 antibody (yellow) and the oligodendroglial markers (A) CC1 (green) and Sox10 (red) or (B) CAII (green) and PDGFRα (red). White boxes (A, B) highlight magnifications of the cerebral cortex and the corpus callosum. (A) C9C5/CC1/Sox10 triple positive cells (white arrowhead) with stellate morphology are presented in merge and single channels with the nuclear marker DAPI. (B) C9C5^+^ (white arrowhead), CAII^+^ (orange arrow), and PDGFRα^+^ (white arrow) cells are presented in merge and single channels with the nuclear marker DAPI. Note that C9C5 positive cells are CAII and PDGFRα negative. Staining was replicated at least on three mice. Ctx, cerebral cortex; cc, corpus callosum.

Immunofluorescent labeling with C9C5 revealed high immunoreactivity throughout most brain regions including the cerebral cortex, the hippocampus, in fiber tracts, in thalamic nuclei, in the tuberal region of the hypothalamus including the median eminence (ME) (Fig 1B and 1C) and in the cerebellum (S2A and S2B Fig). Specificity of C9C5 binding was provided by the absence of these signals in blocking experiments (Fig 1C). Interestingly, C9C5-immunoreactive cells displayed a stellate morphology with intense signal in their cell bodies and their processes that were decorated by immunoreactive puncta (Figs 2A–2C, 3A, 3B, 5E, 6B–6E; S2B, S3A and S3B Figs). These cells were distributed in almost all brain regions analyzed (Figs 1, 2A–2C, 3A, 3B, 5A, 5E and 6D; S2A, S2B, S3A and S3B Figs). In the cerebellum, Purkinje cells are expressing Shh [2, 3] but were C9C5 negative (S2A and S2B Fig) suggesting the antibody does not recognize neuronal Shh.

All C9C5^+^ cells were also immunoreactive for the three oligodendroglial markers Olig2, Sox10 and CC1 (Fig 2A–2C; Fig 3A, S3A Fig). C9C5^+^ cells were not immunoreactive for Platelet-derived growth factor receptor-alpha (PDGFRα), a marker of oligodendrocyte progenitor cells (OPCs) [20] and for carbonic anhydrase (CA) II, a marker of a subpopulation of mature oligodendrocytes [21] (Fig 3B, S3B Fig). Quantification of the distribution of C9C5^+^ cells in these various oligodendroglial cell populations in the hypothalamus and in the cerebral cortex are presented in Table 1. Thus, in these two regions, C9C5^+^ cells represented 11–12% of CC1^+^ cells and 3–6% of Sox10^+^ and Olig2^+^ cells. These data suggest that C9C5 labels a subpopulation of mature oligodendrocytes as shown in hypothalamic nuclei, the ME, the hippocampal and cerebral cortical layers and in the white matter of the corpus callosum (Figs 2A–2C and 3A; S3A Fig). In the hippocampus, these oligodendroglial cells were scattered over the pyramidal cell populations (Fig 2B, red square), were present in the hilus region and the neurogenic niche associated to the granule cell layer of the dentate gyrus (Fig 2B, yellow square; S3A and S3B Fig).

**Table 1:** Quantification of C9C5^+^ cells in oligodendrocyte populations in the adult mouse brain.

	C9C5^+^ cells in oligodendrocyte population (%)	
Marker	Hypothalamus	Cerebral cortex
PDGFRα	0	0*
Olig2	5 ± 1	6 ± 1
Sox10	4 ± 1	3 ± 1
CC1	12 ± 2	11 ± 2
CAII	0	0**

Data are from experiments presented in Figs 2 and 3 and S3 Fig and are means ± SEM from 3–4 mice.

* One C9C5^+^ cell / 364 PDGFRα^+^ cells quantified

** one C9C5^+^ cell / 982 CAII^+^ cells quantified.

**Fig 4:**
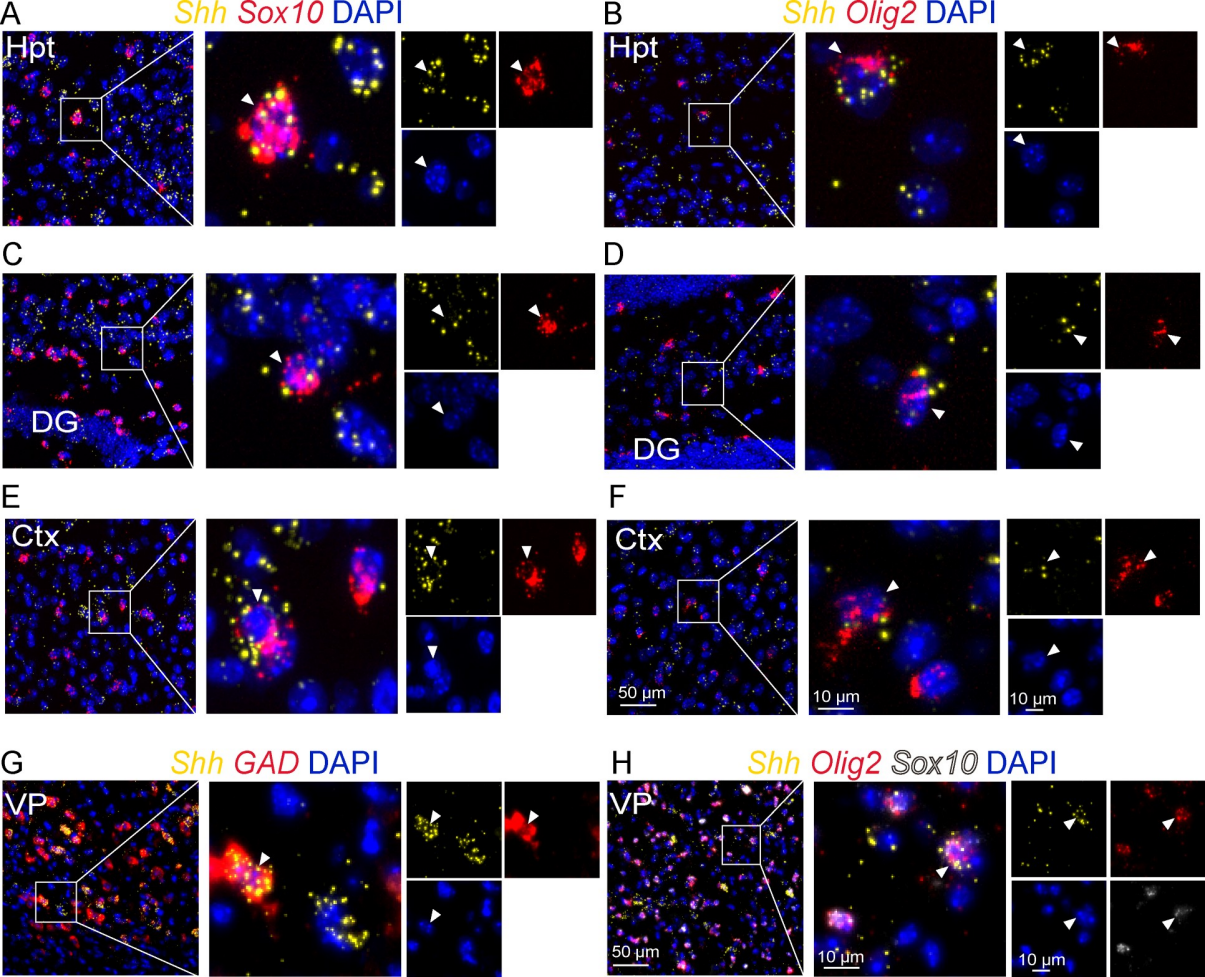
Shh mRNA expression in oligodendroglial cells. Multiplex *in situ* hybridization for Shh and for the oligodendroglial markers Olig2 and Sox10 as indicated, at the level of the tuberal region of the hypothalamus (Hpt) (A-B), the CA3 region of the hippocampus (C-D), the cerebral cortex (Ctx) (E-F), the ventral pallidum (VP) (H), and for the GABAergic marker GAD in the ventral pallidum (G). White boxes highlight magnifications of double Shh/Sox10 (A, C, E), Shh/Olig2 (B, D, F), Shh/GAD (G) or triple Shh/Sox10/Olig2 (H) positive cells (white arrowhead) presented in merge and single channels with the nuclear marker DAPI. Staining was replicated on three mice. DG, dentate gyrus of the hippocampus.

To further support that oligodendroglial cells are a source of Shh in the adult brain, we used single molecule multiplex fluorescent *in situ* hybridization to investigate Shh mRNA distribution. We successfully detected robust expression of Shh transcripts using RNAscope in a subset of scattered cells expressing the transcripts of Olig2 and Sox10 in the brain regions described above (Fig 4A–4F, Table 2). In forebrain regions, Shh mRNAs were detected in glutamic acid decarboxylase (*GAD*)^+^ GABAergic neurons (Fig 4G) as reported previously [10] and in *Sox10*^*+*^*Olig2*^*+*^ oligodendroglial cells (Fig 4H).

**Table 2:** Analysis of Shh mRNA expression in Olig2- and Sox10-expressing cells in the adult mouse brain.

Region	Shh^+^Olig2^+^/Olig2^+^ (% cells)	Shh^+^Sox10^+^/Sox10^+^ (% cells)
Hypothalamus	20.1 ± 3.1	32.3 ± 0.6
Cerebral cortex	12.0 ± 1.3	13.5 ± 0.9

Data are from RNAscope^®^ experiments (means ± SEM, n = 3 mice)

### Shh from C9C5^+^ cells is localized next to Ptc-expressing cells

**Fig 5:**
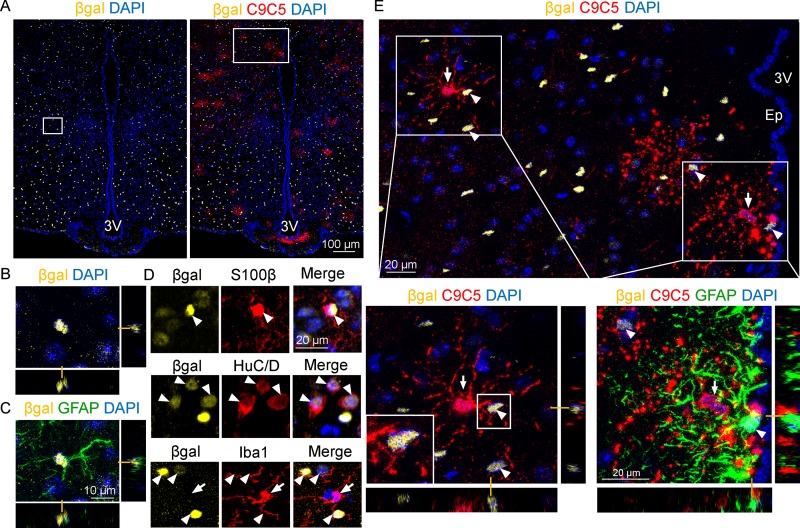
C9C5^+^ cells delineate Shh expression domains containing Ptc-expressing cells. (A-E) Fluorescence confocal (A-C, E) imaging of βgalactosidase (βgal), C9C5, GFAP, S100β, HuC/D, and Iba1 immunostaining on coronal brain sections of adult Ptc^LacZ/+^ mouse hypothalamus at the level of the median eminence. (A, E) Detection of βgal signal (yellow) alone and together with C9C5 signal (red) indicated wide distribution of Ptc-expressing cells in hypothalamic nuclei. (B-C) Orthogonal views of the magnified white square (A, left) showing co-localization of βgal and the astroglial marker GFAP. (D) High magnifications of the hypothalamic parenchyma from independent experiments showing colocalization of βgal (white arrowheads) with the astroglial marker S100β or with the neuronal marker HuC/D, but not with the microglial marker Iba1 (white arrow). (E) White square magnification (from A, right) showing C9C5^+^ cell bodies (white arrows) delineating Shh expression domains in the parenchyma and next to the ependymal layer (Ep) of the third ventricle (3V). White boxes highlight magnifications with orthogonal views of C9C5^+^ processes contacting βgal^+^ Ptc-expressing cells (white arrowheads). Staining was replicated on four mice. Nuclei stained with DAPI.

The distribution of the C9C5 signal in the oligodendroglial cell body and processes suggested that the associated Shh protein could be secreted to act locally on cells expressing its receptor Patched (Ptc). In the tuberal region of the hypothalamus of Ptc^LacZ/+^ knock-in reporter mice, βgalactosidase (βgal) was strongly expressed in GFAP^+^ (Fig 5A–5C) or S100β^+^ (79.3 ± 3.1%, n = 4 animals) (Fig 5D) astrocyte nuclei. βgalactosidase was also detected with a lower intensity in cells positive for the neuronal marker HuC/D but was not identified in cells expressing the microglial marker Iba1 (Fig 5D). Using confocal microscopy, we showed that C9C5^+^ cells and their processes were in close contact with cells showing an intense βgal staining (Fig 5E). These observations suggest that Shh secreted from the oligodendroglial cell could act on several neighboring astrocytes or neurons expressing Ptc.

### Distribution of C9C5^+^ cells varies with age

**Fig 6:**
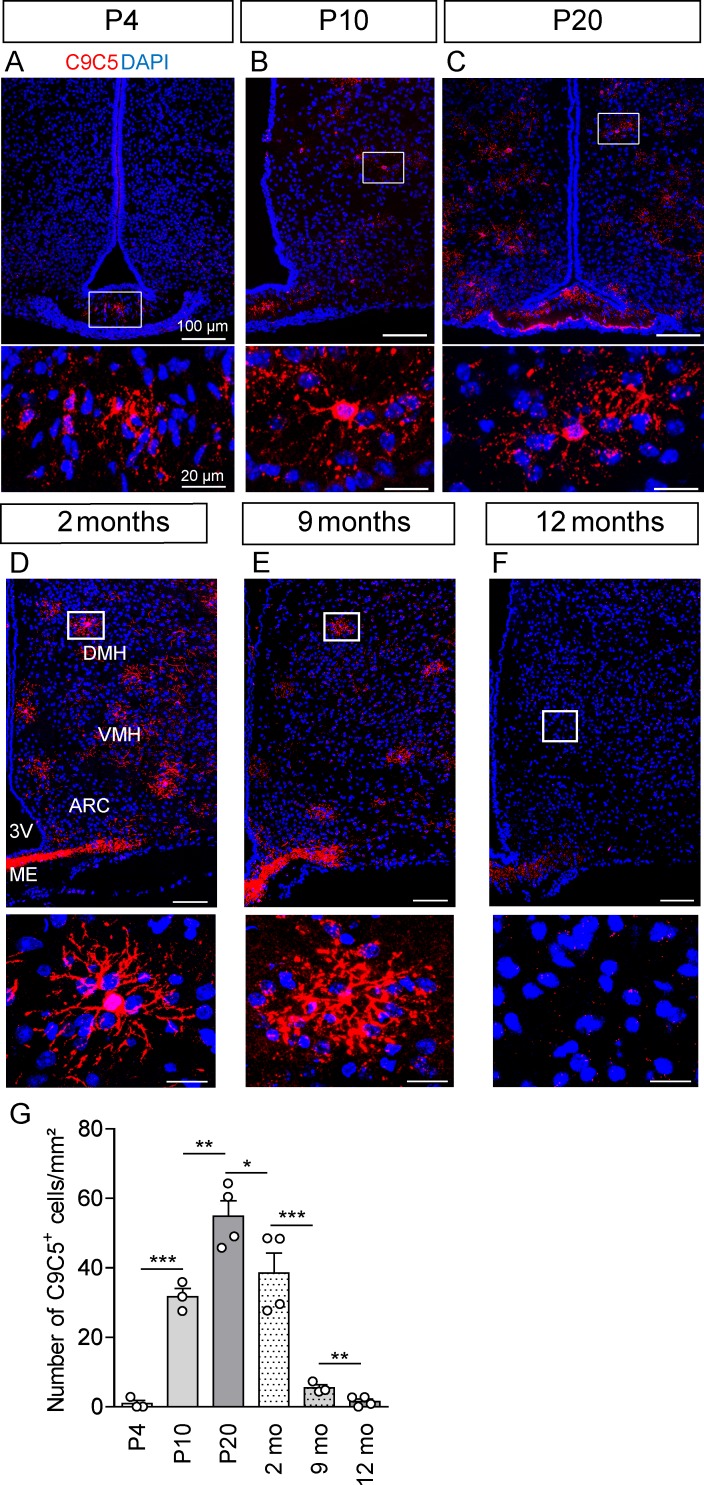
C9C5 immunoreactivity in the mouse hypothalamus across life. (A-F) Immunostaining with the C9C5 antibody on coronal sections of the hypothalamus at P4 (A) (n = 3), P10 (B) (n = 3), P20 (C) (n = 4), and at two (D) (n = 4), nine (E) (n = 3) and twelve (F) (n = 4) months. C9C5^+^ cells are detected in hypothalamic nuclei at P10 until adulthood but not at P4. An age-associated decrease of C9C5^+^ cells in the parenchyma but not in the median eminence (ME) was observed (D-F). Magnifications of white boxes show C9C5^+^ cells with a stellate morphology, which were absent at P4 (A) and in twelve month-old animals (F). C9C5^+^ cells are present in the ME at P4 (A). (G) Quantification of C9C5^+^ cells across life (n = 3–4 per age). Means ± SEM. * p < 0.05; ** p < 0.01; *** p < 0.001. Nuclei stained with DAPI. 3V, third ventricle; mo, months.

Then, we investigated the presence of C9C5^+^ cells in the brain during the postnatal period and in aged animals (Fig 6A–6G). In hypothalamic nuclei, C9C5 immunoreactivity was present in cells displaying stellate morphology starting at P10 with a pattern of cell distribution at P20 that mimicked the adult distribution (Fig 6B–6E). The number of C9C5^+^ cells strongly decreased during ageing in the hypothalamus (Fig 6D–6G). In the ME, C9C5^+^ cells were already observed at P4 (Fig 6A) while intense signal was present in adults and persisted in old animals (Fig 6D–6F), suggesting a different regulation of the signal over time compared to the nearby hypothalamic parenchyma. Quantification of the distribution of C9C5^+^ cells with age in hypothalamic nuclei are presented in Fig 6G.

## Discussion

Here, we report the widespread distribution pattern of the C9C5 immunoreactivity in the adult mouse brain and show that C9C5 antibody marks a subset of CC1^+^ (11–12%), Olig2^+^ and Sox10^+^ (3–6%), CAII^-^ mature oligodendrocytes as quantified in the cerebral cortex and in the hypothalamus. These cells do not express the PDGFRα signal implicated in OPCs proliferation and in the inhibition of the expression of oligodendroglial maturation markers such as CC1 [18, 20]. Thus, these mature oligodendroglial cells constitute a source for delivering Shh to brain cells. Our experiments show that the C9C5 antibody recognizes ShhN in rodent brain extracts as previously reported [22, 23], and on postnatal mouse brain sections. These data are also in agreement with previous reports describing that C9C5 detects specifically Shh in ventral midbrain progenitor domains and in the diencephalic organizer zona limitans intrathalamica of mouse embryo [24, 25], and in adult mouse sensory neurons [26].

Interestingly, we also identify for the first time Shh mRNAs in Olig2^+^ and Sox10^+^ cells in the adult mouse brain using RNAscope which indicates that Shh is more widely expressed than originally reported [10, 14], and suggests novel physiological functions for this molecule. Thus, altogether, our data support that C9C5 recognizes Shh produced by a small population of mature oligodendrocytes. It is not the case for Shh from Purkinje cells of the cerebellar cortex [27, 28] reflecting possible difference in Shh post-translational modifications [1]. We cannot exclude that C9C5 may recognize another yet unknown protein showing SHHN homology, restricted to a subset of oligodendrocytes and which is not Desert Hedgehog or Indian Hedgehog that are not expressed in the rodent brain [2, 27].

Studies using other Shh antibodies [13, 14, 29] or reporter mouse lines [9, 14, 30] did not report Shh expression in oligodendroglial cells in normal adult mouse brain. However, a dynamic expression of Shh in PDGFRα^+^ and Olig2^+^ oligodendrocyte progenitors occurs during remyelination [29]. This apparent discrepancy with our data may reflect different sensitivity of the reporters, of the mouse line constructs or of the antibodies used. For the latter, it may indicate different lipid modifications of ShhN [1] that might occur in oligodendrocyte precursor cells and mature oligodendrocytes.

Interestingly, the Shh signal labels the oligodendroglial cell body and decorates by intense puncta the processes extending at several cell diameters. Thus, Shh could be secreted to act locally on its receptor Ptc expressed by neighboring astrocytes and neurons. It is noteworthy that C9C5^+^CC1^+^ cells are distributed in a grid-like manner with cells residing mostly in non-superposed domains. They constitute small Shh-expressing units that could distribute locally the protein in almost all brain regions and could represent a source for spreading Shh. Thus, our data suggest that besides neurons in the adult forebrain and cerebral cortex [9, 31] and in the cerebellar cortex [8], oligodendroglial cells are a potential source of Shh for signaling in astrocytes and neurons. The appearance of C9C5^+^ and CC1^+^ cells in the postnatal mouse brain overlaps the myelination peak that occurs between P10 and P20 [18] further arguing for a role of mature oligodendrocytes in postnatal Shh pathway regulation.

Recently, an unsuspected oligodendrocyte heterogeneity has been reported by RNA sequencing of oligodendrocyte lineage cells both in the juvenile and adult mouse brain [32, 33]. Further studies of human brain from patients with multiple sclerosis or from spinal cord of mouse models of multiple sclerosis, revealed several clusters of oligodendroglial cells that could be further classified according to their gene expression pattern [34, 35]. It would be important to identify to which clusters C9C5^+^CC1^+^ cells belong and to characterize their properties in CNS inflammation and during remyelination [36]. Thus, the oligodendroglial C9C5^+^Shh-associated signal should undoubtedly be taken into consideration when analyzing brain disorders associated to Shh signaling including brain tumors, neurodevelopmental disorders and neurodegenerative diseases [2].

## Supporting information

S1 Fig Western blot analysis of Shh protein with C9C5 with short and long exposure time. Twenty μg of RIPA solubilized samples from cerebellum (Cb) and hypothalamus (Hpt) and 1μg of mock or mShh transfected HEK293 homogenates were subjected to Western blot and immunodetection with C9C5 antibody (1/1000). Chemiluminescence was acquired with Chemidoc apparatus. The exposure time is indicated below. The 22 kDa (ShhN, filled arrowhead) and 47 kDa (uncleaved Shh; empty arrowhead) bands are indicated. A single band corresponding to ShhN is detected in brain extracts after short and long time exposure, further supporting specificity of the C9C5 antibody.
(TIF)

S2 Fig Characterization of C9C5 immunoreactivity in the adult mouse cerebellum. **(A-B)** Immunostaining with C9C5 and CC1 antibodies on a coronal section of the cerebellum. (B) Magnification of red box indicates that Purkinje cell bodies (white arrowheads), and their projections in the molecular layer are not labeled by the C9C5 antibody. Magnification of white box highlights a C9C5 positive cell expressing the oligodendroglial marker CC1 (white arrow) within the granular cell layer. Staining was replicated on three mice Mol, molecular cell layer; Gr, granular cell layer; Px, Purkinje cells. Scale bar: A, 100 μm. B, 50 μm.
(TIF)

S3 Fig Characterization of C9C5^+^ cells at different stages of the oligodendroglial lineage. (A-B) Immunostaining of coronal brain sections from an adult mouse of the hypothalamus at the level of the median eminence and of the dentate gyrus of the hippocampus with the C9C5 antibody (yellow) and the oligodendroglial markers (A) CC1 (green) and Sox10 (red) or (B) CAII (green) and PDGFRα (red). White boxes (A, B) highlight magnifications of the hypothalamic parenchyma and the granule cell layer of the dentate gyrus. (A) C9C5/CC1/Sox10 triple positive cells (white arrowhead) with stellate morphology are presented in merge and single channels with the nuclear marker DAPI. (B) C9C5^+^ (white arrowhead), CAII^+^ (orange arrow), PDGFRα^+^ (white arrow) cells are presented in merge and single channels with the nuclear marker DAPI. Note that C9C5 positive cells are CAII and PDGFRα negative. Staining was replicated on three mice. Hpt, hypothalamus; DG, dentate gyrus of the hippocampus.
(TIF)

S1 Raw images (PDF)
