# Supplementary figures and images for: C9C5 positive mature oligodendrocytes are a source of Sonic Hedgehog in the mouse brain
Source: PLoS One. 2020 Feb 20;15(2):e0229362. doi: 10.1371/journal.pone.0229362 (PMC7032736; doi:10.1371/journal.pone.0229362)

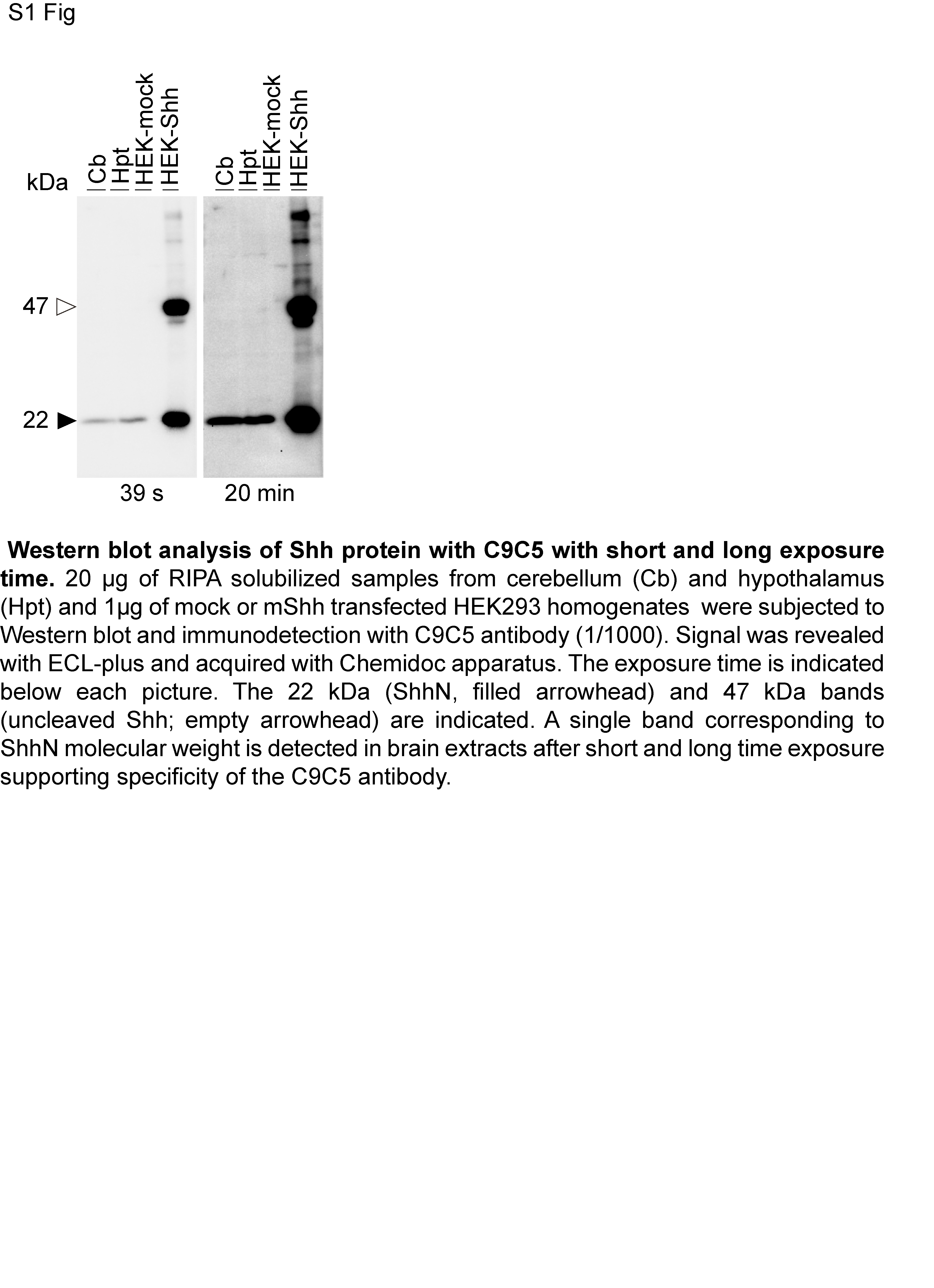

Supplement: S1 Fig — Twenty μg of RIPA solubilized samples from cerebellum (Cb) and hypothalamus (Hpt) and 1μg of mock or mShh transfected HEK293 homogenates were subjected to Western blot and immunodetection with C9C5 antibody (1/1000). Chemiluminescence was acquired with Chemidoc apparatus. The exposure time is indicated below. The 22 kDa (ShhN, filled arrowhead) and 47 kDa (uncleaved Shh; empty arrowhead) bands are indicated. A single band corresponding to ShhN is detected in brain extracts after short and long time exposure, further supporting specificity of the C9C5 antibody. (TIF) [file pone.0229362.s001.tif]

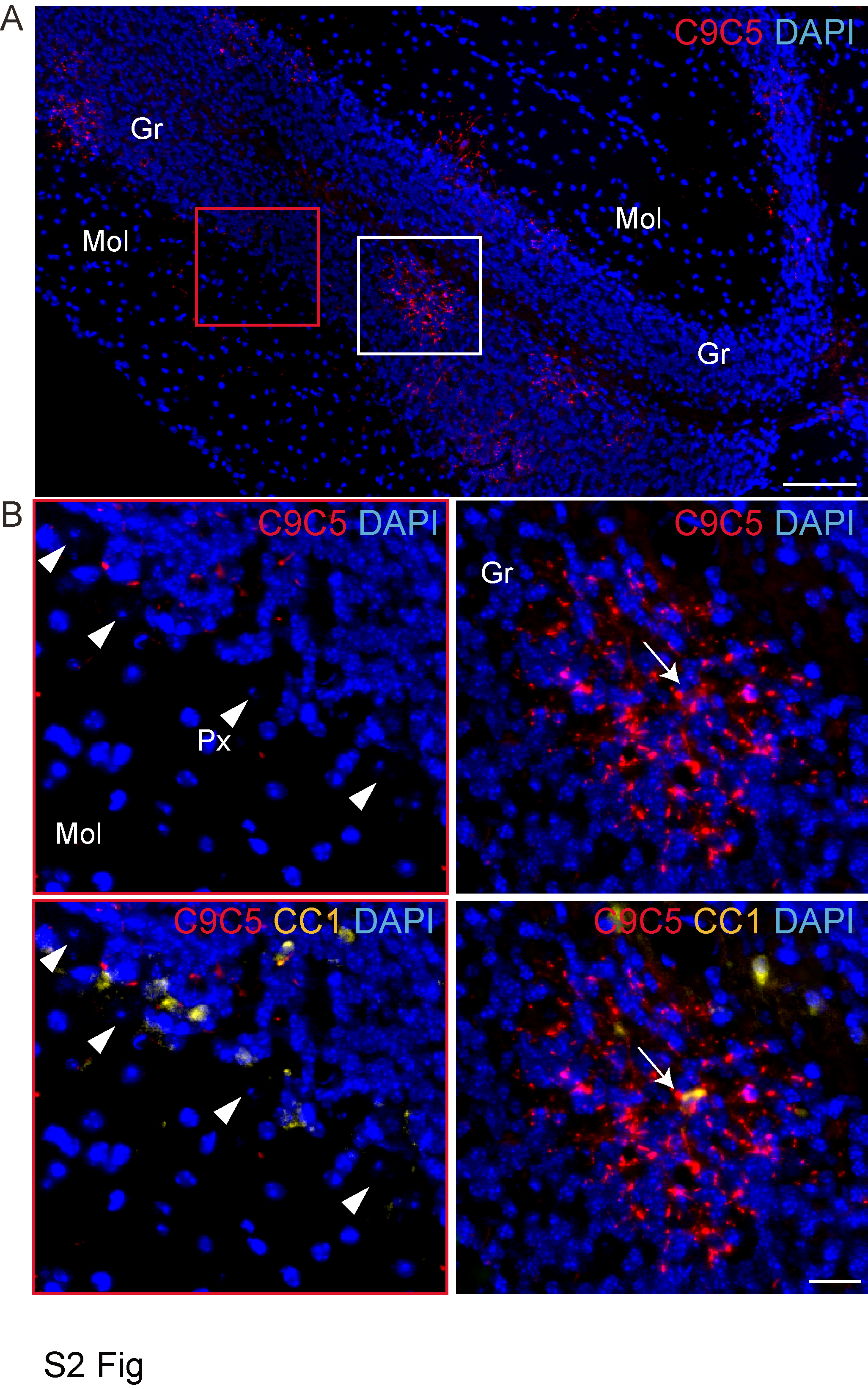

Supplement: S2 Fig — (A-B) Immunostaining with C9C5 and CC1 antibodies on a coronal section of the cerebellum. (B) Magnification of red box indicates that Purkinje cell bodies (white arrowheads), and their projections in the molecular layer are not labeled by the C9C5 antibody. Magnification of white box highlights a C9C5 positive cell expressing the oligodendroglial marker CC1 (white arrow) within the granular cell layer. Staining was replicated on three mice Mol, molecular cell layer; Gr, granular cell layer; Px, Purkinje cells. Scale bar: A, 100 μm. B, 50 μm. (TIF) [file pone.0229362.s002.tif]

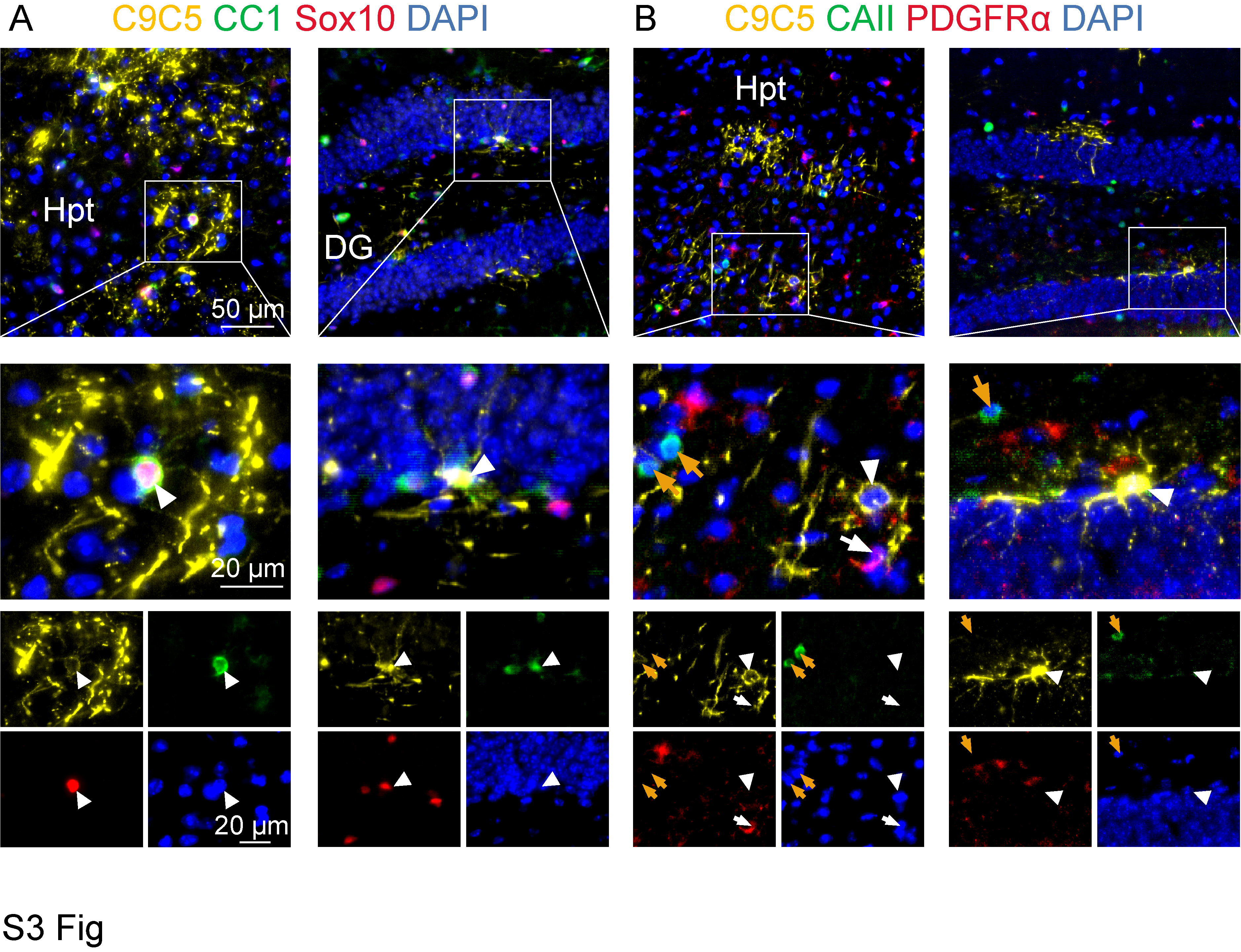

Supplement: S3 Fig — (A-B) Immunostaining of coronal brain sections from an adult mouse of the hypothalamus at the level of the median eminence and of the dentate gyrus of the hippocampus with the C9C5 antibody (yellow) and the oligodendroglial markers (A) CC1 (green) and Sox10 (red) or (B) CAII (green) and PDGFRα (red). White boxes (A, B) highlight magnifications of the hypothalamic parenchyma and the granule cell layer of the dentate gyrus. (A) C9C5/CC1/Sox10 triple positive cells (white arrowhead) with stellate morphology are presented in merge and single channels with the nuclear marker DAPI. (B) C9C5+ (white arrowhead), CAII+ (orange arrow), PDGFRα+ (white arrow) cells are presented in merge and single channels with the nuclear marker DAPI. Note that C9C5 positive cells are CAII and PDGFRα negative. Staining was replicated on three mice. Hpt, hypothalamus; DG, dentate gyrus of the hippocampus. (TIF) [file pone.0229362.s003.tif]
