# Supplementary material for: C9C5 positive mature oligodendrocytes are a source of Sonic Hedgehog in the mouse brain
Source: PLoS One. 2020 Feb 20;15(2):e0229362. doi: 10.1371/journal.pone.0229362 (PMC7032736; doi:10.1371/journal.pone.0229362)

Uncropped gels, from Image lab software for Figure1A, Tirou et al. PlosOne

Left part of Figure 1A

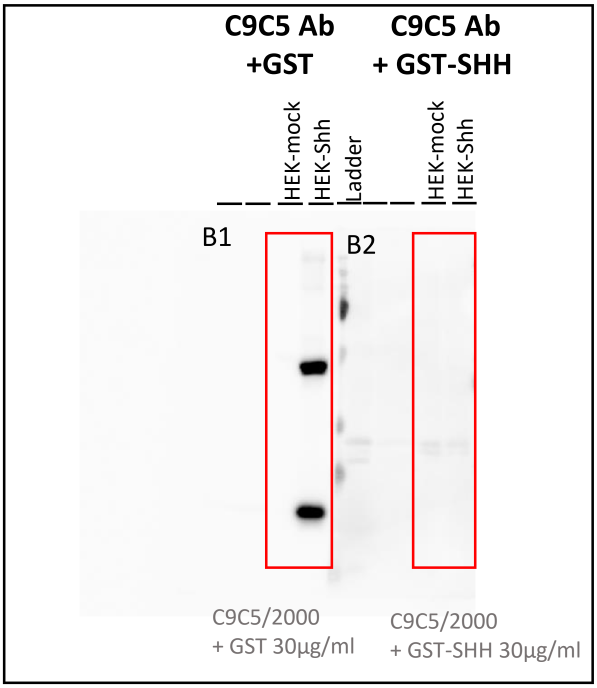

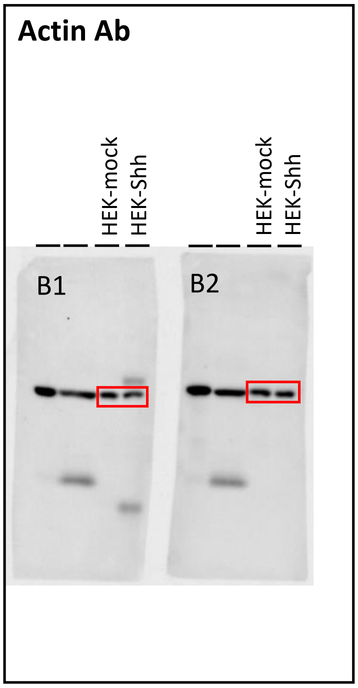

Right part of Figure 1A

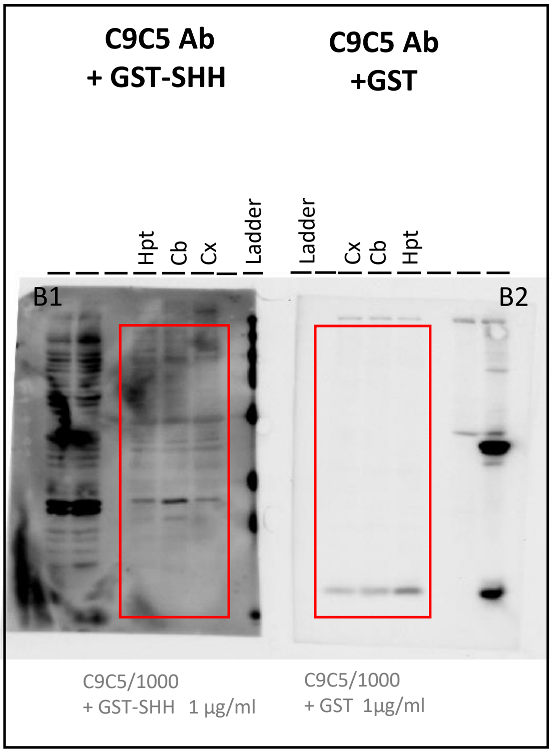

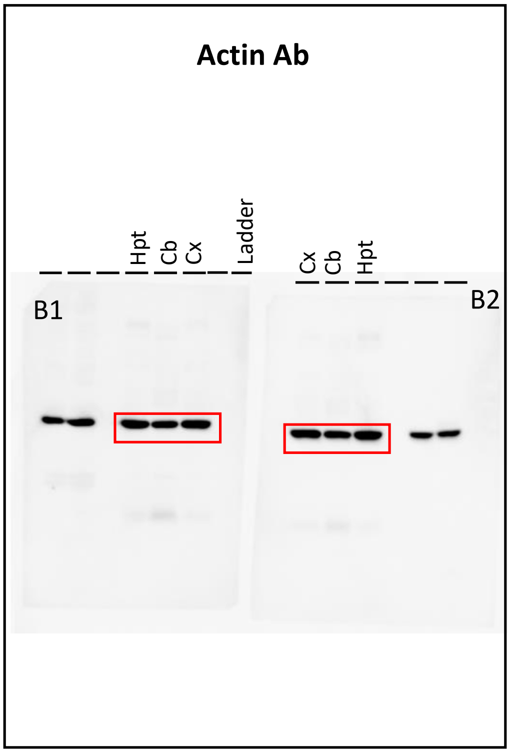

Uncropped gels, from Image lab software for Figure S1, Tirou et al. PlosOne

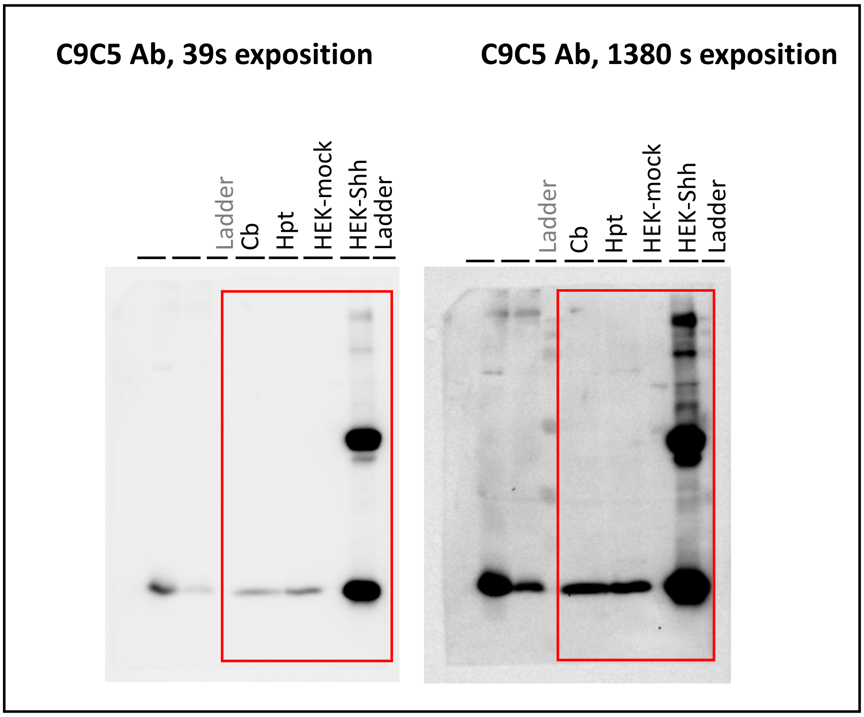

Supplement: S1 Raw images — (PDF) [file pone.0229362.s004.pdf]
